# Exploring drivers of patient satisfaction using a random forest algorithm

**DOI:** 10.1186/s12911-021-01519-5

**Published:** 2021-05-13

**Authors:** Mecit Can Emre Simsekler, Noura Hamed Alhashmi, Elie Azar, Nelson King, Rana Adel Mahmoud Ali Luqman, Abdalla Al Mulla

**Affiliations:** 1 Department of Industrial and Systems Engineering, Khalifa University of Science and Technology, P.O. Box 127788, Abu Dhabi, UAE; 2 Mubadala Healthcare, Ipic Square, Abu Dhabi, 45005 UAE

**Keywords:** Patient satisfaction, Quality, Healthcare operations, Patient experience, Random forests, Data analytics, Machine learning

## Abstract

**Background:**

Patient satisfaction is a multi-dimensional concept that provides insights into various quality aspects in healthcare. Although earlier studies identified a range of patient and provider-related determinants, their relative importance to patient satisfaction remains unclear.

**Methods:**

We used a tree-based machine-learning algorithm, random forests, to estimate relationships between patient and provider-related determinants and satisfaction level in two of the main patient journey stages, registration and consultation, through survey data from 411 patients at a hospital in Abu Dhabi, UAE. Radar charts were also generated to determine which type of questions—demographics, time, behaviour, and procedure—influence patient satisfaction.

**Results:**

Our results showed that the ‘age’ attribute, a patient-related determinant, is the leading driver of patient satisfaction in both stages. ‘Total time taken for registration’ and ‘attentiveness and knowledge of the doctor/physician while listening to your queries’ are the leading provider-related determinants in each model developed for registration and consultation stages, respectively. The radar charts revealed that ‘demographics’ are the most influential type in the registration stage, whereas ‘behaviour’ is the most influential in the consultation stage.

**Conclusions:**

Generating valuable results, the random forest model provides significant insights on the relative importance of different determinants to overall patient satisfaction. Healthcare practitioners, managers and researchers can benefit from applying the model for prediction and feature importance analysis in their particular healthcare settings and areas of their concern.

## Background

### Patient satisfaction

Patient satisfaction has gained significant attention as a critical component of health service quality [1–3]. Patients are regarded as the best candidates for providing vital source of information about the care received, the possible barriers to obtaining care and the providers’ interpersonal behaviour [4, 5]. Earlier studies showed that higher patient satisfaction with health services has positive impacts [1, 6], resulted in better health outcomes and recommendations of the hospital services to others [7].

Although many studies evaluated patient satisfaction in the literature, it still remains difficult to identify the determining factors of this multi-dimensional concept. The concept involves a range of factors varied considerably across the literature [2, 8–12]. Further, there is an absence of an absolute consensus on the theoretical framework of patient satisfaction [13, 14]. Therefore, identifying which set of determinants drive patient satisfaction is still debatable. This great diversity in potential drivers of patient satisfaction leads to multiple dimensions in measurement studies, which reduces the ability to compare them and draw meaningful conclusions [1]. Further, there are limited methodological tools and models measuring patient satisfaction [1, 15].

Surveys are commonly used tools in assessing patient satisfaction [7, 16]. They help capture patient judgments about the received health service [17]. Despite numerous studies that have either developed new surveys to evaluate patient satisfaction or adjusted existing ones [18, 19], further research is required to consider all potential factors, including patient and provider-related determinants [1]. Moreover, the multi-dimensional satisfaction determinants, with possible interactions between each other, and their association with patient satisfaction, might not be well understood in this research context using currently available tools. However, machine learning tools, such as tree-based ensemble learning algorithms, may provide opportunities with feature importance analysis and prediction capabilities to better evaluate patient satisfaction determinants throughout the patient journey.

### Random forests

The use of artificial intelligence (AI) and machine learning (ML) algorithms has gained a growing awareness in various domains and industries [20–22], including the healthcare industry [23, 24]. As a subset of ML, tree-based algorithms also gained particular attention with their realistic and easy-to-interpret results [25–27]. These tools' contribution has been notably recognized with their prediction accuracy and handling interactions in big data sets automatically, even if large covariates are present [25].

As a non-parametric ensemble method, random forests (RFs) has gained popularity in dealing with regression and classification problems [28, 29]. The RFs develops many decision trees [30, 31] using a random subset of variables obtained independently and with replacement from the original dataset [27, 32]. One of the important features of the RFs is the built-in feature importance functionality that helps rank the independent variable regarding their importance to the outcome variable, which adds value in data analysis [27, 33]. Encouraging results in both empirical [34, 35] and theoretical [36] studies have been conducted in various domains, including healthcare [37, 38]. Although RFs have merits in data analytics with various functions on multiple datasets, their implementation is limited in the patient satisfaction context.

### Study aims

This study aims to evaluate patient satisfaction by ranking the importance of patient and provider-related determinants in two of the common patient journey stages, *registration* (check-in) and *consultation* process, using a random forest algorithm. Even though many hospitals have developed programs and patient satisfaction surveys to assess the quality of health services, limited research is available capturing determinants affecting patient satisfaction in different patient journey stages. Therefore, this research is designated to explore this matter using patient satisfaction survey data provided by a hospital in the UAE. The research focuses on understanding patient satisfaction drivers, which fall within four types of questions: demographic, time, procedural, and behavioural related questions. Understanding such drivers will help identify important features; therefore, potential areas for improvement in healthcare quality research and practice.

## Methods

### Data source

In this study, retrospective and de-identified patient satisfaction survey data are collected from a hospital in Abu Dhabi, UAE. In total, 411 patients participated in the survey and were asked to rate their experience throughout their hospital journey. Data from two common journey stages are included in this study: (1) *registration* as a non-clinical stage; and (2) *consultation* as a clinical stage in the patient journey. Each section includes a set of questions that fall into five main categories: (1) *demographics*, such as nationality, gender, age as well as their visit type (e.g., new patient or established patient), (2) *time-related* questions, (3) *behaviour-related* questions, (4) *procedure-related* questions, and a question on (5) *overall satisfaction* in relevant patient journey stages. While demographics represent patient-related determinants, time, behaviour and procedure questions represent provider-related determinants. The questions are mainly five-Likert-style survey items, asking how satisfied patients are and illustrating the following scale: *not at all* (1), *not* (2), *neutral* (3), *somewhat* (4), *extremely* (5).

### Procedure

In this study, the chosen data analysis method is RFs, while employing Python as the programming language. Table 1 describes the libraries used in the proposed algorithm implementation.

**Table 1:** Python libraries used in this study

Library	Description
Pandas	Provides high-level data structures and many more tools [46]
Matplot	Used for data visualization and can create two-dimensional graphs and diagrams [46]
Seaborn	Used for data visualization with extensive settings for processing charts [47]
Scikit-learn	Exposes many of the machine learning packages [48]

Two models, Model 1 and Model 2, are developed for each patient journey stage included in this study. There is one target variable (overall patient satisfaction) and several explanatory variables in each model. Both models follow the same development and testing process.

In each model, we first identify hyper-parameters, such as *max depth*, *n estimators*, *minimum samples split* and *minimum samples leaf*, used commonly in RF algorithms [27, 39]. Table 2 provides the hyper-parameters with short descriptions and ranges obtained from a recent study [20].

**Table 2:** Parameter search space in grid search analysis

Parameter	Range
max_depth (maximum depth of the tree)	[3; 4; 5; 6; 7; 8; 9; 10; 12; 15]
min_samples_leaf (minimum number of data points allowed in a leaf node)	[1; 2; 3; 4; 5; 6; 7; 8; 9; 10; 15]
min_samples_split (minimum number of data points in a node before the node is split)	[2; 3; 4; 5; 6; 8; 10; 15]
n_estimator (number of trees in the forest)	[100; 150; 200; 250; 300; 400; 500; 600; 800; 1000; 1200]

The grid search with a cross-validation method is applied to select the optimal parameter combinations for the tuning process. The *k*-fold cross-validation method is then used to split the training set (80% of the sample) into *k* number of subsets, known as folds. In this study, the training set is divided into five folds to be evaluated. The model is trained using the first four folds and validated with the fifth fold. In the second iteration, the training is repeated on the second, third, fourth, and fifth folds and evaluated on the first fold. This procedure is repeated five times so that each time the evaluation is on a different fold. Following this, the scores from each run are averaged, and the optimal model is identified. By averaging out all the validation scores, an optimal model is attained [33]. Following this, we assess the model's predictive performance solely on the test set (20% of the sample) to reduce potential bias [20]. Using Eq. 1 below, the accuracy metric is obtained for each model developed.

1 $$Accuracy = \frac{number\;of\;correct\;predictions}{{total\;number\;of\;records}}$$

As an essential component of RFs, feature importance is obtained to visually represent each feature's relative importance (also known as determinant) on the trained model [33]. The importance of each feature is calculated by Mean Decrease in Impurity (MDI), described as the total reduction in node impurity averaged over all ensemble trees [27].

Following the RFs, radar charts are generated to determine which of four question types—*demographics, procedure, behaviour,* and *time*—most influence patient satisfaction according to the weighted average of the feature importance scores.

## Results

Table 3 summarizes the responses to the patient-related determinant questions in percentages. Of the sample, 89.1% were locals, while the remaining 10.9% were foreigners. More male than female respondents filled out the survey (54.3% and 45.7%, respectively). Patients over the age of 65 constituted 34.6% of admitted patients. New patients comprised 78.4% of respondents.

**Table 3:** Patient-related determinants

Patient-related determinants		Percentage
1.1_Nationality	Locals	89.1
	Foreigners	10.9
1.2_Gender	Male	54.3
	Female	45.7
1.3_Age	Age group 1 (<21)	7.3
	Age group 2 (21 to 30)	22.9
	Age group 3 (30 to 40)	22.1
	Age group 4 (40 to 50)	34.6
	Age group 5 (50 to 65)	12.7
	Age group 6 (>65)	0.5
1.4_PTN	New patient	78.4
	Established patient	21.7

Following the descriptive results, a reliability analysis was conducted to verify the internal consistency in time, behaviour, and procedure-related questions. Results showed that the Cronbach’s alpha of each determinant type in each patient journey stage is above 0.9, providing validation for the specific variables' aggregation into a single type of question.

### Prediction results

Table 4 illustrates the hyper-parameter values used in each model along with the average accuracy score. We measured the accuracy values as 0.78 and 0.93 in the *registration* and *consultation* stages, respectively. In light of these results, it can be said that the models are capable of predicting the test data.

**Table 4:** Results of random forest models

Performance metric/hyper-parameters	Model 1: registration process	Model 2: consultation process
Accuracy	0.78	0.93
Hyper-parameters		
*max_depth*	10	10
*min_sample_leaf*	2	2
*min_sample_split*	2	7
*n_estimators*	100	150

In the *registration* model, the best possible hyper-parameter combination consists of 100 estimators, two samples as the minimum requirement to split an internal node, two samples as the minimum for a leaf node, and a maximum depth of 10. In the *consultation* model, the optimal value for estimators is 150; seven samples were required as the minimum for splitting an internal node. Two samples were the minimum for a leaf node, and the maximum depth was 10.

### Feature importance analysis

**Fig. 1:**
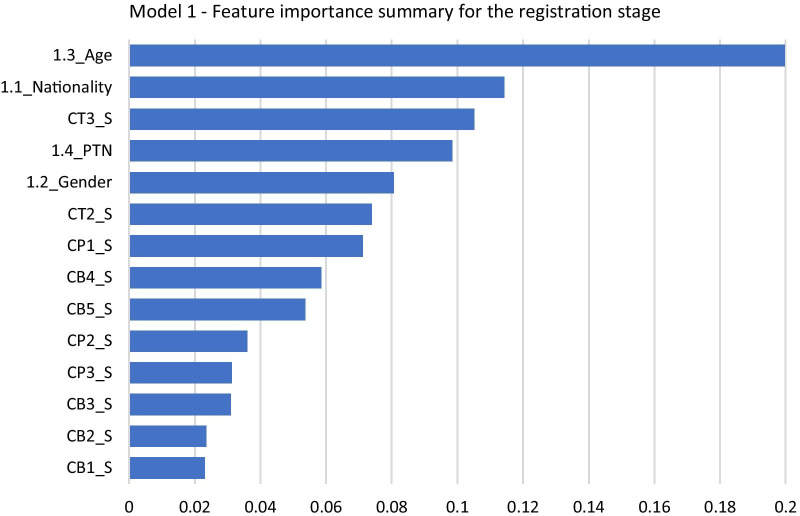
Model 1: feature importance summary for the registration stage

After training the algorithm and optimizing the model, the variables' feature importance scores were plotted for Model 1 and Model 2. Figure 1 demonstrates the feature importance ranking for the registration model. The *y*-axis represents the explanatory variables, while the *x*-axis represents the feature importance score. The explanatory variables—i.e., the survey questions relevant to the registration—are ranked from highest to lowest. In the registration stage, the “1.3_Age” variable obtained the highest score in this model, followed by the variable “1.1_Nationality,” which also belongs to patient-related determinants. The third important variable in this model is “CT3_S,” which refers to the survey question “Total time taken for registration” and belongs to the provider-related determinants category. The “1.4_PTN” and “1.2_Gender” variables also have high feature importance scores.

These results indicate that patient-related determinants are among the top five drivers in this model, emphasizing their importance in driving patient satisfaction in the registration stages. The variables “CB2_S” and “CB1_S”—which correspond to the survey questions “Professionalism of the registration staff’s appearance” and “Approachable and smiling manner of the registration staff,” respectively—had the lowest feature importance score.

Table 5 shows that two of the three main drivers among provider-related determinants are related to the “time” question type in the registration stage, while none are related to the “behaviour” type.

**Table 5:** Top three drivers of patient satisfaction in the registration stage

Determinants	Rank	Importance score	Question code	Survey question
Patient-related determinants	1st	0.20	1.3_Age	Age group
	2nd	0.11	1.1_Nationality	Nationality
	3rd	0.10	1.4_PTN	Patient type (e.g., new patient and established patient)
Provider-related determinants	1st	0.11	CT3_S	Total time taken for registration (time-related)
	2nd	0.07	CT2_S	Time taken upon arrival to acknowledge you at the registration desk (time-related)
	3rd	0.07	CP1_S	Knowledge of the registration staff whilst handling the registration process (procedure-related)

Using a radar chart, we used the feature importance score results and clustered them to understand which type of questions (i.e., demographic, time, procedure, or behaviour) has the greatest influence. The value was obtained by finding the average of all feature importance scores corresponding to all variables belonging to each question, then dividing that average by the sum of the averages of all question types.

**Fig. 2:**
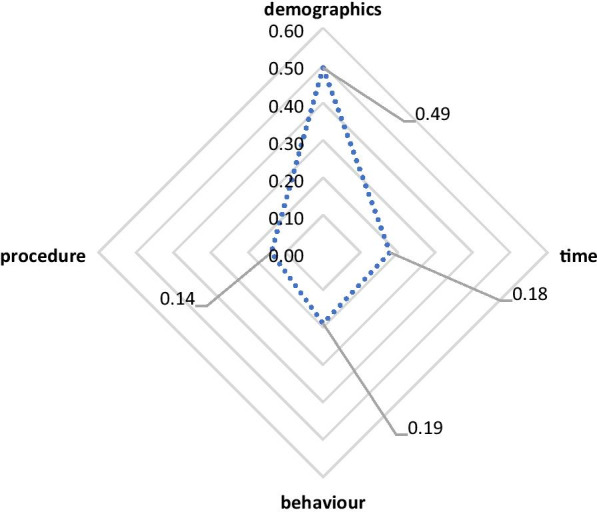
Radar chart for the registration stage

Figure 2 illustrates the importance score for each question type in the *registration* model. The results showed that “demographics” was the dominant question type in the *registration* model, as it had the highest importance score, followed by the “time” question type.

**Fig. 3:**
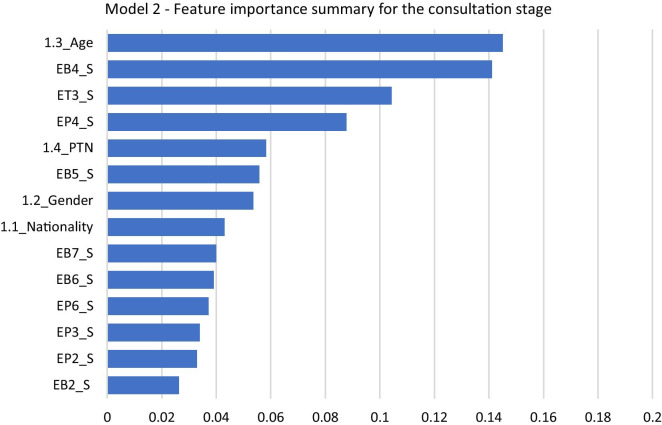
Model 2: feature importance summary for the consultation stage

Figure 3 illustrates the feature importance for the *consultation* model. Similar to the registration model, “1.3_Age” is the top-ranked variable here. The second most important variable for this model was “EB4_S,” which corresponds to “Attentiveness and knowledge of the doctor/physician while listening to your queries,” followed by “ET3_S,” which corresponds to “Waiting time to see the doctor/physician.” The “EP4_S” variable, corresponding to “Doctor/physician’s explanation of the next steps in treatment (e.g., tests, medications, etc.),” was of moderate importance. Unlike the previous model, patient-related determinant attributes were not the leading drivers in this model. The variable which least contributed to patient satisfaction in this model is “EP1_S,” which corresponds to the “Doctor’s level of awareness of previously collected data (history and physical)” survey question.

Table 6 shows the top three drivers of patient-related determinants. However, two of these variables—“1.4_PTN” and “1.2_Gender”—contribute less to patient satisfaction levels, as their feature importance scores are relatively low. The leading drivers among provider-related determinants are from different question types (*behaviour*, *time* and *procedure*).

**Table 6:** Top three drivers of patient satisfaction in the consultation stage

Determinants	Rank	Importance score	Question code	Survey question
Patient-related determinants	1st	0.14	1.3_Age	Age group
	2nd	0.06	1.4_PTN	Patient type (e.g., new patient and established patient)
	3rd	0.05	1.2_Gender	Gender
Provider-related determinants	1st	0.14	EB4_S	Attentiveness and knowledge of the Doctor/Physician while listening to your queries (behaviour-related)
	2nd	0.10	ET3_S	Waiting time to see the Doctor/Physician (time-related)
	3rd	0.09	EP4_S	Doctor/Physician’s explanation of the next steps in treatment (e.g., tests, medications, etc.) (procedure-related)

**Fig. 4:**
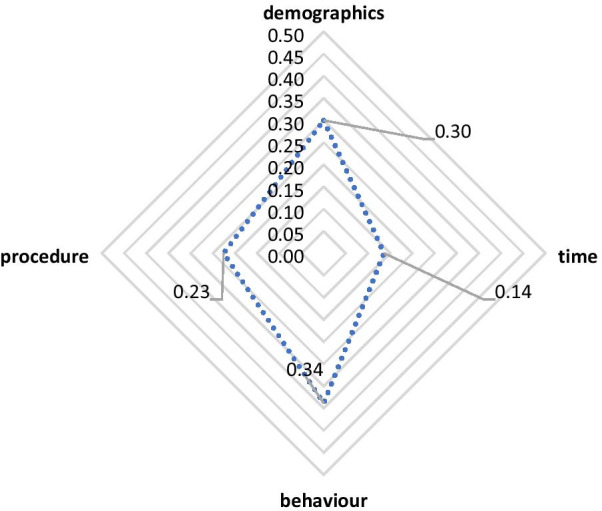
Radar chart for the consultation stage

Figure 4 shows the radar chart for the *consultation* stage. The results show that the “behaviour” question type has the highest feature importance score, making it the most influential type of question in the *consultation* stage, followed by the “demographics” and “procedure” types. This indicates the critical role of provider-related determinants in the *consultation* stage compared to the *registration* stage.

After demonstrating our results and analysis, it is important to discuss the findings using the existing literature. The following section, therefore, discusses our results in further detail.

## Discussion

In this study, the random forest algorithm was developed and used to identify the main drivers contributing to patient satisfaction in each patient journey stage.

In the model developed for the *registration* stage, the most important attributes were patient-related determinants and belonged to the “demographics” question type, as indicated in the radar chart. The variable “1.3_Age”, which refers to the patient's age, was the most important of all variables belonging to the “demographics” question type, based on the feature importance scores. This result is reasonable since age is considered one of the most important factors influencing patient satisfaction [40].

Based on the feature importance scores, “1.4_PTN”—which refers to whether a patient is a new or established patient—was the third most important attribute in the *registration* model. This attribute's importance in influencing patient satisfaction was also found in another study, which showed that frequent visits to the hospital have a potential effect on patient satisfaction [41]. Further, the “1.1_Nationality” attribute was the second most important attribute in the *registration* model, which indicates that this attribute influences patient satisfaction. These findings are consistent with earlier research showing that factors such as age and nationality are associated with patient satisfaction [42].

In the *consultation* model, the radar chart showed that the most influential type of question is the “behaviour” type. This indicates that, in this clinical stage, provider-related determinants belonging to the “behaviour” question type were most important and influenced patient satisfaction. Most of the time, patients recommend a medical facility to others according to physicians' affective behaviours [43]. According to the feature importance score, the survey question “Attentiveness and knowledge of the doctor/physician while listening to your queries” (considered a “behaviour” question) was most important in the provider-related determinant category. This finding is consistent with earlier findings that a provider’s communication skills, listening skills, and nonverbal communication skills are positively associated with patient satisfaction [1]. This emphasizes the crucial role of providers’ affective behaviours in dealing with their patients.

In the *registration* model, patient-related determinant attributes were dominant, as mentioned earlier. In this model, the first and second most important variables are “Total time taken for registration” and “Time taken upon arrival to acknowledge you at the registration desk,” respectively. These two variables are also “time” questions. Having two variables that belong to time questions as top drivers in the *registration* model is not surprising. Earlier research has shown a tight correlation between wait time and patient satisfaction [6, 44]. Long waiting times, which may result from inefficient use of available capacity or poor design of services, are associated with decreased patient satisfaction [44, 45]. The third most important variable in the *registration* model is “Knowledge of the registration staff whilst handling the registration process,” which is also procedure-related.

In the *consultation* model, the attributes in the “Patient-related determinant” category did not play a significant role in patient satisfaction. However, the variables in the provider-related determinant category that belonged to the “behaviour” question category were dominant. According to the feature importance scores, the most important variable is “Attentiveness and knowledge of the doctor/physician while listening to your queries”. As pointed out in the literature, healthcare professionals' perceived competency is linked with patient satisfaction [1]. The second most important variable was “Waiting time to see the doctor/physician,” which is a “time” question. Finally, the third most important variable is the “Doctor/physician’s explanation of the next steps in treatment (e.g., tests, medications, etc.),” which is a “procedure” question. The role of healthcare professionals is essential in providing support and information. Therefore, their competence in providing treatment options and decisions may be linked with patient satisfaction [1].

The results of this section can be summarized in two main observations. First, the attributes relevant to patient-related determinants and belonging to the “demographics” question type were dominant in the *registration* stage. This is due to the high feature importance score of the attributes included in the “demographics” type of question. The “1.3_Age” attribute was ranked first in the *registration* model, which indicates the importance of such an attribute and its influence on patient satisfaction. Second, the most influential type of question in the *consultation* process is “behaviour”, which falls in the provider-related determinant category. The variable that was ranked first here was “Attentiveness and knowledge of the doctor/physician while listening to your queries,” highlighting the importance of the role of providers’ communication skills in dealing with patients.

The RF algorithm developed in this study provided significant insight on feature importance in both patient journey stages. It should be noted that the RF algorithms, like many other machine learning algorithms, are open to improvement through tuning parameters to provide better accuracy. Although we applied grid search analysis for tuning, future studies may benefit more from thorough optimization of the hyper-parameters to identify their best possible combination to provide more accurate predictions. Future studies can also evaluate supervised and unsupervised algorithms to explore their accuracy in predicting patient satisfaction drivers.

This study has several limitations. As survey data is specific to a particular hospital, the RF results' transferability may be limited to other hospitals and medical institutions. Therefore, the generalizability of our results to other healthcare settings also remains unclear. Future studies may benefit from using a similar method to perform prediction and feature importance analysis with their patient satisfaction survey data.

## Conclusions

In this research, we presented survey analysis results to understand the key drivers of patient satisfaction in two of the typical patient journey stages. The RF models captured important limitations in the existing literature on patient satisfaction determinants as most of the earlier studies tackled an insufficient number of determinants without reflecting their relative importance to satisfaction. Further, the algorithm addressed the complex relationship between the variables. The random forest classifier was used to identify the different determinants of patient satisfaction. The algorithm was implemented and validated on patient satisfaction survey data containing responses from 411 patients from a hospital in Abu Dhabi, UAE. The key findings of the applied analysis can be summarized in the following points:

- “Age” attribute, a *patient-related determinant*, was the leading driver of patient satisfaction in all models according to its high feature importance score.
- “Total time taken for registration” and “Attentiveness and knowledge of the Doctor/Physician while listening to your queries” were the *provider-related determinants* driving patient satisfaction in each model.
- The radar charts revealed that “demographics” was the most influential type of question in the *registration* model, while “behaviour” was the most influential in the *consultation* model.

The main contribution of this study is to identify and rank the patient satisfaction drivers in two patient journey stages. Another contribution of this study is the development of a novel ML algorithm using patient satisfaction survey data. The results can provide hospitals significant insights into the different determinants affecting patient satisfaction. To our knowledge, this is the first study using RFs in the patient satisfaction context. We conclude that the RFs algorithm exhibited predictive capability and shed light on the relationship between specific determinants and overall patient satisfaction. Healthcare organizations invest significant resources to improve patient satisfaction. The results of the study may help them prioritize resource usages based on the importance ranking to achieve sustainable improvements in the patient satisfaction context.

## Data Availability

Contact the corresponding author who can connect interested parties to the data holder.

## Abbreviations

RF: Random forest
ML: Machine learning
AI: Artificial intelligence
